# Prevalence of misconception about HIV/AIDS transmission and associated factors among reproductive age women in Ethiopia: a nationwide study

**DOI:** 10.1186/s12879-023-08884-8

**Published:** 2024-01-06

**Authors:** Ali Yimer, Abdul-Aziz Kebede Kassaw, Sebwedin Surur, Endris Mussa

**Affiliations:** 1 Department of Public Health, College of Health Sciences, Woldia University, Woldia, Ethiopia; 2 Department of Health Informatics, School of Public Health, College of Medicine and Health Sciences, Wollo University, Dessie, Ethiopia; 3 Department of statistics, College of natural and computational sciences, Mizan Tepi University, Mizan Tepi, Ethiopia; 4 Department of Software Engineering, College of Informatics, Wollo University, Kombolcha, Ethiopia

**Keywords:** Misconceptions, HIV transmission, Women, Ethiopia

## Abstract

**Background:**

Misconceptions and myths are still the bottlenecks for the prevention of HIV/AIDS transmission in developing countries. This study aimed to assess the prevalence and associated factors of misconception about HIV transmission among reproductive age groups using the most recently available Ethiopian Demographic and Health Surveydata.

**Methods:**

A cross-sectional study design was done using the Ethiopian Demographic and Health Survey 2016 data set. The data analysis was conducted using  Statistical Package for Social Sciences version 25. Multivariable logistic regression analysis was done to identify associated factors of misconception about HIV/AIDS transmission. A *p*-value of < 0.05 and an adjusted odds ratio with a 95% confidence interval were considered to confirm a statistically significant association.

**Results:**

From the sample of 11,425 reproductive-age women, the prevalence of misconception about HIV/AIDS transmission among reproductive-age women in Ethiopia was 27.47%. Women residing in rural area [AOR:1.24; 95% CI: 1.03–1.75] compared to urban resident participants, attended primary education [AOR:0.58;95%CI: 0.49–0.68], attended secondary education [AOR:0.36;95%CI:0.29–0.46], attended higher education [AOR:0.24;95%CI: 0.18–0.32] compared to those participants without education, had history of HIV test [AOR:0.77; 95%CI: 0.67–0.88] compared to their counterpart, respondents living in Amhara region [AOR:0.44:95% CI:0.35–0.54], Benishangul [AOR: 0.34; 95% CI: 0.25–0.46], SNNPR [AOR:0.50; 95% CI: 0.38–0.67], Gambela [AOR:0.57; 95% CI: 0.42–0.79], Harari [AOR:0.62; 95% CI: 0.46–0.82], Addis Ababa [AOR:0.63; 95% CI: 0.49–0.81] compared to those living in Tigray and having richest wealth status[AOR:0.57;95% CI: 1.457–4.078] compared to those whose wealth index was poorest were significantly associated with the misconception about HIV transmission.

**Conclusion:**

Over all the prevalence of misconception about HIV/AIDS transmission among reproductive-age women in Ethiopia was high. Residence, educational level, wealth index, region, and respondents who ever tested for HIV were significantly associated with the misconception about HIV/AIDS transmission. This high misconception could affect HIV/AIDS transmission and its prevention strategies unless timely and appropriate intervention should be taken. Strengthening strategies aimed at maximizing HIV/AIDS testing, scaling up educational status, and emphasizing regional-wide interventions might have a substantial contribution.

## Background

HIV infection is responsible for the most devastating human pandemics. It has created a seriously painful challenge worldwide. HIV has infected close to 70 million people, and more than 30 million have died due to acquired immunodeficiency syndrome (AIDS) since its recognition. Of the total 40 million people living with HIV/AIDS, more than 66% of them are in sub-Saharan Africa, where AIDS is the leading cause of death [1]. Ethiopia is the second most populous and one of the seriously affected countries in sub-Saharan Africa. More than 1.3 million people living with HIV and an estimated 277,800 people requiring treatment, despite the Government of Ethiopia has taken measures to reduce the risk of HIV transmission and mitigate the impact of the epidemic on society [1].

HIV/AIDS has been known as one of the main challenging public health concern in Ethiopia since the mid-1980 [2]. Being fatal and attacking the most productive as well as reproductive age group are the typical devastating features of the disease [3, 4]. Now a days, the effects of HIV/AIDS are not merely a public health issue but also affect the entire day-to day social and economic living conditions of people [5].According to the 2020 country fact sheet report on HIV/AIDS(UNAIDS). Ethiopia has a total of 620,000 people living with HIV. Of these,360,000 were women of childbearing age with 8900 adults newly infected [6].

Studies showed that blood transfusions, venous injections with contaminated needles, unsafe sexual intercourse, and vertical transmission from mother to child are the most common ways of HIV/AIDS transmission [7].However; misconceptions could lead to incorrect information and results in incorrect attitudes about HIV/AIDS mode of transmission [8]. Despite scaling-up behavioral change mechanisms about HIV/AIDS transmission, misconception about its transmission is among the vital prevalent problems in developing countries including Ethiopia [9].

According to a study conducted in rural china revealed that 70.4, 24, 24.6, 26.5, and 41.8% of women perceived that mosquito bites, talking with an HIV infected person, handshaking, food sharing, and swimming respectively were assumed to be route of HIV/AIDS transmission [10]. Similarly, 39% of female respondents perceived that mosquito bites could transmit HIV/AIDS, according to a study conducted in Botswana [8]. Moreover, according to a study conducted in India, 85.3 and 79.3% of the study participants reported that HIV/AIDS could be transmitted through food sharing and means of supernatural force respectively [11]. And, based on the Ethiopian Demographic and Health Survey (EDHS) 2016 report, 70% of women perceived that HIV can be transmitted by using food together with the infected person and through mosquito bites [12].

Likewise, reproductive-age women suffered from the devastating risk of HIV due to various barriers and inequalities like poverty, increased risk of sexual violence, cultural inequities, economic disempowerment, traditional beliefs, and gender power imbalance in sexual interactions [13].

Misconceptions could be a major challenge to retard the burden of the disease and its interventional activities [14, 15]. A study conducted in United States of America showed that a misconception regarding HIV/AIDS transmission could expose to various risky behaviors and it leads to a delay to take intervention against its spread [16].

Based on the findings of various studies, different factors are associated with a misconception about HIV transmission; of which residing in rural areas [7, 17], lack of education [7, 17], health illiteracy [18], religion [9], wealth index [9], occupation [13] and media access were the typical factors [13].

Myths and misconceptions are still being the barriers for the strategies of HIV transmission reduction in developing countries despite the delivery of health information related to HIV transmission being sustained across different countries [13]. Previous research findings indicated that misconception endorsement about HIV transmission is connected to risky sexual behaviors like unsafe sexual intercourse and unable to use preventive mechanisms [19, 20].

Despite the reproductive age groups are the most vulnerable targeted age groups which need research priority, there is little information in Ethiopia regarding the prevalence and associated factors of misconception about HIV transmission for the desire of such targeted productive generations. Therefore; this study aims to fill such a prominent gap by assessing the prevalence and associated factors of misconceptions about HIV transmission among reproductive age women using the most recently available demographic and health surveys of Ethiopia.

## Methods

### Study design and setting

A cross-sectional study design was conducted among reproductive age women in Ethiopia using the 2016 Ethiopian Demographic and Health Survey data.

### Sample size estimation and sampling technique

**Fig. 1:**
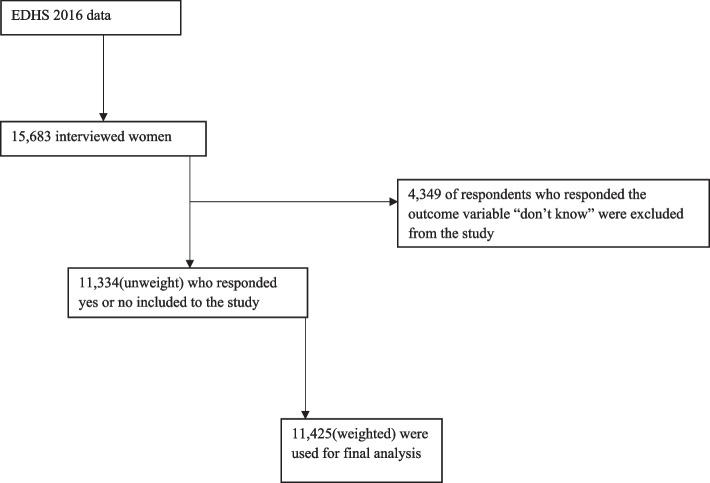
Sampling procedure for women’s misconceptions about HIV transmission

The study used the national set of representative data extracted from the EDHS 2016 [21]. In the 2016 Ethiopian Demographic and Health Survey data, a total of 18,008 households for the sample were selected. The total household size was 16,650 and of these 15,683 were eligible women [12]. Among the interviewed women, 15,683 responded to the interview. Among 15,683, only 4349 respondents who responded, “don’t know” were excluded from the study. Finally, 11,334 unweight or 11,425 weighted samples (“In DHS surveys, in most surveys the sample is selected with unequal probability to expand the number of cases available (and hence reduce sample variability) for certain areas or subgroups for which statistics are needed. In this case, weights need to be applied when tabulations are made of statistics to produce the proper representation”) were included in this study (Fig. 1).

The samples were selected using a two-stage stratified cluster sampling technique. Each region was stratified into urban and rural areas, yielding 21 sampling strata. Samples of EAs were selected independently in each stratum in two stages. In the first stage, a total of 645 EAs (202 in urban areas and 443 in rural areas) were selected with a probability proportional to each EA size. In the selection of the second stage, a fixed number of 28 households per cluster were selected with an equal probability selection from the newly created household listing. All women who had an age group between15–49 who were either permanent residents of the selected households or visitors who stayed in the household at night before the survey were eligible to be interviewed [22] (Fig. 1).

### Dependent and independent variables

Outcome variable in this study was misconception about HIV/AIDS transmission.

**Explanatory variables**: social demographic variables like region, age, sex, marital status, educational level, place of residence, and wealth index and behavioral variables like occupational status, media access, and ever been tested for HIV were explanatory variables in this study.

### Operational definition

**Outcome variable:** The unit of analysis of the study was the misconception about HIV ADIS transmission. To assess misconception about HIV transmission among reproductive-age women, 3 misconception-related indicators were selected from the EDHS 2016 data [21]. The indicators were as follows: (i) can get HIV from mosquito bites? (ii) Can get HIV by sharing food with a person who has AIDS? And (iii) can get HIV by witchcraft or supernatural means? The respondents answered in a trichotomies form as (i) no, (ii) yes, and (iii) do not know. These indicators were subsequently categorized in the dichotomous form as (1) no misconception, if the respondents answer” No″ for all the three questions and (2) yes(misconception), if the respondents answer at least one yes and finally remove the last category (do not know) [23].

### Data quality control

The data was from 2016 EDHS secondary data. The data was extracted by following strictly required procedures as shown in the above figure one. Data cleaning was done. EDHS 2016 quality of the data was determined primarily by the quality of the fieldwork, following appropriate steps that can enhance it considerably during data processing. Data entry and editing for inconsistencies were critical steps in this research to remove missing data.

### Data processing and analysis

Frequencies and weighted percentages of the variables were presented to explain the profile of the study participants. Due to the binary (dichotomous) nature of the outcome variable, we applied binary logistic regression analysis. The analysis was done using Statistical Package for Social Sciences (SPSS) version 25. Adjusted odds ratio (AOR) had been used to report the analysis results. Multi-variable logistic regression was conducted on independent predictors by adjusting the covariates. A *p*-value of < 0.05 and 95% confidence intervals were used to declare a statistically significant association. Sample weighting was applied for adjustment of cluster sampling design and sampling probabilities through strata and clusters. Model fitness was assessed by Hosmer and Lemeshow goodness of fit test, which showed a *p*-value of greater than 0.05.

## Results

### Socio-demographic characteristics of study participants

A total of 11,425 reproductive-age women were included in this study. Five thousand five hundred fifty three (48.6%) women were ever been tested for HIV.Out of a total study participants (Table 1), 7015(61.4%) reproductive-age women were married. 4463 (39.06%) study participants had a primary educational level. About 40.7% of the respondents were tested for HIV. Moreover, 41.8% of the respondents had media access (Table 2). The prevalence of misconception about HIV/AIDS transmission among reproductive-age women in Ethiopia in this study was 27.47%.

**Table 1:** Socio demographic profile of Reproductive Age Women in Ethiopia using 2016 EDHS data (*n* = 11,425)

Variable	Category	Misconception about HIV transmission			
		No		Yes	
		Count	%	Count	%
Respondent’s current age	15–24	2880	46.5%	1992	38.1%
	25–34	1941	31.3%	1815	34.7%
	35–44	1072	17.3%	1085	20.8%
	45–49	306	4.9%	333	6.4%
Region	Tigray	423	6.8%	476	9.1%
	Afar	35	0.6%	51	1.0%
	Amhara	1596	25.7%	895	17.1%
	Oromia	1646	26.5%	2317	44.3%
	Somali	69	1.1%	81	1.6%
	Benishangul	84	1.3%	39	0.7%
	SNNPR	1658	26.7%	1121	21.4%
	Gambela	23	0.4%	11	0.2%
	Harari	20	0.3%	10	0.2%
	Addis Ababa	603	9.7%	195	3.7%
	Dire Dawa	43	0.7%	29	0.6%
Type of place of residence	Urban	2155	34.8%	831	15.9%
	Rural	4044	65.2%	4395	84.1%
Religion	Orthodox	2935	47.3%	2016	38.6%
	Catholic	55	0.9%	46	0.9%
	Protestant	1709	27.6%	1276	24.4%
	Muslim	1454	23.5%	1809	34.6%
	Others	47	0.8%	78	1.5%
Highest educational level	No education	1779	28.7%	2684	51.4%
	Primary	2521	40.7%	1942	37.2%
	Secondary	1229	19.8%	459	8.8%
	Higher	671	10.8%	140	2.7%
Marital status	single	1751	28.2%	1526	29.2%
	married	3817	61.6%	3198	61.2%
	Separated/divorced	632	10.2%	501	9.6%
Wealth status	Poorest	590	9.5%	947	18.1%
	Poorer	874	14.1%	1018	19.5%
	Middle	986	15.9%	1106	21.2%
	Richer	1264	20.4%	1063	20.3%
	Richest	2485	40.1%	1091	20.9%

**Table 2:** Behavioral profile of Reproductive Age Women in Ethiopia using 2016 EDHS data (*n*=11425)

Variable	Category	Misconception about HIV transmission			
		No		Yes	
		Count	%	Count	%
Respondent currently working	No	3865	62.3%	3556	68.1%
	Yes	2335	37.7%	1669	31.9%
Ever been tested for HIV	No	2775	44.8%	3097	59.3%
	Yes	3425	55.2%	2128	40.7%
Media access	No	2724	43.9%	3039	58.2%
	Yes	3476	56.1%	2186	41.8%

Key: others: Waqiefeta and bete Israel

### Association factors of misconception about HIV/AIDS transmission among reproductive age women in Ethiopia

According to the binary logistic regression analysis results (Table 3), residence, educational level, wealth index, region, and respondents who ever tested for HIV were significantly associated with a misconception about HIV transmission in the study area.

**Table 3:** Bivariable and multivariable analysis of misconception about HIV/AIDS transmission among reproductive-age women in Ethiopia using 2016 EDHS data (*n* = 11,425)

Variable	COR [CI 95%]	AOR [CI 95%]
Age		
15–24	1	1
25–34	1.35 [1.19, 1.54]*	1.13 [0.96, 1.33]
35–44	1.46 [1.27, 1.69]	1.05 [0.89, 1.24]
45–49	1.57 [1.24, 1.99]	1.06 [0.82, 1.37]
Region		
Tigray	1	1
Afar	1.31 [0.87, 1.98]	0.92 [0.65, 1.32]
Amhara	0.50 [0.39, 0.64]***	0.44*** [0.35, 0.54]
Oromia	1.25 [0.95, 1.65]*	1.09 [0.85, 1.41]
Somali	1.05 [0.76, 1.45]	0.72 [0.50, 1.03]
Benishangul	0.41 [0.31, 0.55]***	0.34*** [0.25, 0.46]
SNNPR	0.60 [0.46,0.78]***	0.50 ***[0.38, 0.67]
Gambela	0.44 [0.33,0.59]***	0.57 ***[0.42, 0.79]
Harari	0.46 [0.34, 0.61]***	0.62 ***[0.46, 0.82]
Addis Ababa	0.29 [0.23, 0.36]***	0.63*** [0.49, 0.81]
Dire Dawa	0.61 [0.45, 0.82]*	0.90 [0.66, 1.22]
Residence		
Urban	1	1
Rural	2.82 [2.37, 3.36]**	1.24* [1.03, 1.75]
Religion		
Orthodox	1	1
Catholic	1.22 [0.69, 2.22]*	1.03 [0.49, 2.17]
Protestant	1.09 [0.90, 1.31]	0.88 [0.70, 1.12]
Muslim	1.81 [1.47, 2.23]	0.99 [0.80, 1.23]
Others	2.40 [1.56, 3.66]	0.95 [0.49, 182]
Educational level		
No education	1	1
Primary	0.51 [0.45, 0.58]***	0.58*** [0.49, 0.68]
Secondary	0.25 [0.21, 0.30]***	0.36 ***[0.29, 0.46]
Higher	0.14 [0.11, 0.18]***	0.24*** [0.18, 0.32]
Marital status		
Single	1	1
Married	0.96 [0.83, 1.12]*	0.91 [0.79, 1.04]
Separated	0.91 [0.75, 1.11]	0.86 [0.67, 1.07]
Wealth		
Poorest	1	1
Poorer	0.73 [0.58, 0.91]***	0.77*** [0.62, 0.96]
Middle	0.70 [0.55, 0.89]***	0.82 ***[0.64, 1.05]
Richer	0.52 [0.42, 0.66]***	0.69 ***[0.55, 0.86]
Richest	0.27 [0.22, 0.34]***	0.57 ***[0.43, 0.74]
Working status		
No	1	1
Yes	0.78 [0.69, 0.88]	0.99 [0.88, 1.12]
Ever tested HIV		
No	1	1
Yes	0.56 [0.49, 0.64]***	0.77*** [0.67, 0.88]
Media access		
No	1	1
Yes	0.56 [0.49, 0.65]*	1.10 [0.92, 1.23]

Odds ratio adjusted for clustering; 95% confidence intervals in brackets

*: *p* < 0.05;**: *p* < 0.01; ***: *p* < 0.001; 1: reference group: others: Waqiefeta and bete Israel

Holding the effect of other covariates constant, a misconception about HIV transmission for those respondents who had lived in Amhara Region decreased by 56% than those who had lived in Tigray region. Furthermore, the misconception about HIV transmission for those who had lived in Benishangul, SNNPR, Gambela, Harari, and Addis Ababa decreased by 66, 50, 43, 38 and 37% than those who had lived in Tigray region respectively.

Moreover, misconception about HIV transmission decreased by 43% among the richest respondents than those who were the poorest.

Likewise; respondents who lived in a rural area were 1.24 times [95% CI: 1.03–1.75] more likely to have a misconception about HIV transmission than their counterparts.

Furthermore, a misconception about HIV transmission decreased by 23% among respondents who had a history of HIV test than those respondents who did not have a history of HIV test.

Additionally; misconception about HIV transmission decreased by 42, 64, and 76% among respondents who had a primary school, secondary school, and higher institution educational level respectively than respondents who didn’t attend education at all.

## Discussion

We examined the prevalence of a misconception about HIV/AIDS transmission and associated factors among reproductive-age women in Ethiopia. In this study, the prevalence of misconception about HIV/AIDS transmission was 27.47%, which was significantly associated with residence, educational level, wealth index, region, and respondents who ever tested for HIV.

This finding showed that respondents who were living from Harari, Benishangul, SNNPR, Gambela, and Amhara regions showed decreased misconception about HIV/ AIDS transmission than respondents who were living in Tigray region. This result is consistent with the study conducted in Yujiro, Malawi, which identified that Tumbuka’s women were more likely to have a misconception about HIV/AIDS transmission than women from other Malawi ethnicity groups [24]. This might be due to the similarity in sample size, socioeconomic status, and population lifestyle characteristics.

Moreover, respondents who had higher educational levels showed lower misconception about HIV/AIDS transmission as compared to those who did not have formal education. This finding is consistent with a study conducted on Malawi’s general population [9]. This similarity might be due to the nationwide nature of the study and large sample size incorporation. It is also in line with a study conducted in Ghana [25]. This might be due to the reason that those who have attended higher education can get opportunities to identify the typical means of HIV/AIDS transmission and misconception about its transmission. Besides, the finding of this study was in agreement with a study conducted in Bangladeshi (21). This might be due to the similar sex composition of respondents. It is also in line with a study conducted in Malawi. This could be due to the reason that educated respondents have more exposure to knowledge, attitudes, perceptions, and awareness about HIV/AIDS transmission and protection methods [9].

Respondent’s wealth index had a significant association with misconception about HIV/AIDS transmission. The misconception about HIV/AIDS transmission among wealthier respondents decreased compared to their counterparts. This is consistent with previous studies [17, 25]. This is because wealthier respondents have high opportunities to access health information and health education through various media regarding HIV/AIDS transmission and related misconception.

According to this study, misconception about HIV/AIDS decreased among urban residents than who were rural residents. This sighting is in line with a study in Botswana and Bangladesh, which revealed the chance of having misconception increases among rural participants [9, 17, 25]. Rural residents were found to have greater odds of having incomplete knowledge or inappropriate HIV-related attitude and behavior [26]. A similar result was found in a study conducted in Kenya, which showed that rural women reported a higher level of stigmatization. This finding is also consistent with a study conducted by Yebei which showed there was a higher level of non-accepting attitude toward people leaving with HIV in rural residents [26]. This might be due to the higher educational level and its opportunity in urban settings than their counter parts. Respondents who had a history of HIV tests had lower misconception about HIV/AIDS transmission compared with those respondents who did not have a history of HIV tests. This finding is consistent with the study conducted in Malawi [9]. This could be due to those respondents who had a history of HIV tests might acquire good knowledge during counseling services while they came for HIV testing services. Large and nationwide sample usage is the crucial strength of this study; this can enable this study to infer the results for the general population. The limitation of this study was the restriction of researchers just to the variables found in the survey since the research is conducted using secondary data. Another limitation of this study was the use of self-reported information, which may have some potential for reporting biases. Additionally, the analysis was only among reproductive-age women. 

## Conclusions

The prevalence of misconception about HIV/AIDS transmission among reproductive-age women in Ethiopia was high. Residence, educational level, wealth index, region, and respondents who ever tested for HIV were significantly associated with the misconception about HIV/AIDS transmission among reproductive-age women in Ethiopia. This high misconception could have a devastating effect regarding the prevention of HIV/AIDS transmission unless immediate interventions should be taken. Strengthening HIV/AIDS testing and counseling, scaling-up educational status, and regional-specific and targeted interventions might have a paramount roles to alleviate the problem at the grass roots level.

## Data Availability

The datasets used or analyzed during the current study are available from the corresponding author on reasonable request.

## Abbreviations

AIDS: Acquired Immunodeficiency Syndrome
EDHS: Ethiopian Demographic and Health Survey
HIV: Human Immunodeficiency Virus
SPSS: Statistical Packages for Social Sciences
